# Quantitative and dynamic profiling of human gut core microbiota by real-time PCR

**DOI:** 10.1007/s00253-024-13204-4

**Published:** 2024-06-26

**Authors:** Ziheng Yan, Tongyu Hao, Yanfeng Yan, Yanting Zhao, Yarong Wu, Yafang Tan, Yujing Bi, Yujun Cui, Ruifu Yang, Yong Zhao

**Affiliations:** 1 State Key Laboratory of Pathogen and Biosecurity, Beijing Institute of Microbiology and Epidemiology, Beijing, 100071 China; 2 Beijing Key Laboratory of POCT for Bioemergency and Clinic, Beijing, 100071 China

**Keywords:** Gut microbiota, Core microbe, Species abundance, Quantitative methods, Real-time PCR

## Abstract

**Abstract:**

The human gut microbiota refers to a diverse community of microorganisms that symbiotically exist in the human intestinal system. Altered microbial communities have been linked to many human pathologies. However, there is a lack of rapid and efficient methods to assess gut microbiota signatures in practice. To address this, we established an appraisal system containing 45 quantitative real-time polymerase chain reaction (qPCR) assays targeting gut core microbes with high prevalence and/or abundance in the population. Through comparative genomic analysis, we selected novel species-specific genetic markers and primers for 31 of the 45 core microbes with no previously reported specific primers or whose primers needed improvement in specificity. We comprehensively evaluated the performance of the qPCR assays and demonstrated that they showed good sensitivity, selectivity, and quantitative linearity for each target. The limit of detection ranged from 0.1 to 1.0 pg/µL for the genomic DNA of these targets. We also demonstrated the high consistency (Pearson’s *r* = 0.8688, *P* < 0.0001) between the qPCR method and metagenomics next-generation sequencing (mNGS) method in analyzing the abundance of selected bacteria in 22 human fecal samples. Moreover, we quantified the dynamic changes (over 8 weeks) of these core microbes in 14 individuals using qPCR, and considerable stability was demonstrated in most participants, albeit with significant individual differences. Overall, this study enables the simple and rapid quantification of 45 core microbes in the human gut, providing a promising tool to understand the role of gut core microbiota in human health and disease.

**Key points:**

• *A panel of original qPCR assays was developed to quantify human gut core microbes.*

• *The qPCR assays were evaluated and compared with mNGS using real fecal samples.*

• *This method was used to dynamically profile the gut core microbiota in individuals.*

**Supplementary information:**

The online version contains supplementary material available at 10.1007/s00253-024-13204-4.

## Introduction

The human gut microbiota refers to the diverse microbial community that exists symbiotically in the human intestinal system. Mounting evidence suggests that the gut microbiota plays a critical role in human health, including roles in nutrient metabolism, immunity maintenance, and resistance against colonization by exogenous microorganisms (de Vos et al. 2022; Hou et al. 2022). Significant alterations in the composition and structure of the human gut microbiota have been linked to the occurrence and development of various diseases, such as inflammatory bowel disease (IBD), colorectal cancer, obesity, and diabetes (de Wit et al. 2023; Winter and Bäumler 2023; Yang et al. 2021). Changes in the gut microbiota are considered crucial risk factors for these diseases. By identifying altered signatures in the gut microbiota, healthcare professionals can gain valuable insights into various diseases and assess the health status of patients. Therefore, gut microbiota signatures hold great potential as biomarkers for certain disease conditions (Coker et al. 2022; Metwaly et al. 2022; Ning et al. 2023). However, there is a lack of rapid and efficient appraisal systems to accurately quantify microbiota signatures in practice. This significantly limits the widespread clinical application of gut microbiota–based diagnostic and therapeutic methods.

The human gut microbiota is a complex ecosystem comprising trillions of microorganisms (Lawal et al. 2023). Although the composition of the gut microbiota varies significantly among individuals (Daybog and Kolodny 2023), certain microbial members, such as *Bacteroides* (Zafar and Saier 2021) and *Faecalibacterium prausnitzii* (Miquel et al. 2013), are high abundance or prevalence in the general population. Researchers have identified several microbes that have important physiological or ecological functions in gut homeostasis, such as the production of butyrate (*Eubacterium rectale*), modulation of immune responses (*Akkermansia muciniphila*), and reduction of gut microbial lipopolysaccharide production (*Bacteroides vulgatus* and *Bacteroides dorei*) (Bae et al. 2022; Lu et al. 2022; Yoshida et al. 2018). These microbial members are commonly referred to as the gut core microbiota, because they are widely present in healthy individuals and probably the most ecologically and functionally important microbes in the gut (Martínez et al. 2013; Shetty et al. 2017). Although the definition of core microbiota varies depending on the inclusion criteria, such as the occurrence, abundance, or both, it is generally agreed that the core microbiota plays a crucial role in maintaining gut homeostasis and overall health (Neu et al. 2021; Risely and Tate 2020). Researchers recently constructed a defined 119-member community comprising the most prevalent human gut microbes in vitro and found that the colonized community in gnotobiotic mice exhibited robust stability to fecal challenge and strong colonization resistance against pathogenic *Escherichia coli* (Cheng et al. 2022). This study highlights the potential of core microbiota as an in vitro therapy strategy for maintaining intestinal homeostasis and preventing or treating pathogen infections. Given the indispensability of the core microbiota in maintaining gut health, these microbes may serve as indicators for assessing the status of the gut microbiota and might become potential biomarkers for health monitoring and disease diagnosis.

Currently, the most common tool for studying gut ecosystems is metagenomic next-generation sequencing (mNGS) (Swarte et al. 2023). This is a discovery-oriented and culture-independent method that allows the analysis of various microbes in a sample. Although mNGS is an extremely valuable tool in the identification of unknown pathogens and microbiota communities (Wu et al. 2023), it has some considerable limitations for wide clinical application. The most evident limitations are that mNGS usually takes several days and lacks standardization in both sequencing and analysis procedures (Chiu and Miller 2019). In addition, the high cost and requirement of a professional bioinformatics background and vast computing resources further exacerbate the dilemma (Liu et al. 2020; Simner et al. 2018). Given the above, when detecting a specific pathogen of interest or a limited variety of known bacteria in clinical practice, such as these core microbes mentioned above, other more rapid and simple methods are more suitable than mNGS, such as real-time quantitative polymerase chain reaction (qPCR) (Kurina et al. 2020). Compared with mNGS, qPCR is a well-established method for rapid detection and quantification of microorganisms with reliable reproducibility; and it possesses the obvious advantages of simplicity and speed in operation (1–2 h or less) (Nguyen et al. 2022). Both qPCR and mNGS can detect bacteria at concentrations lower than 10^3^ copies/mL (Hasan et al. 2020); however, mNGS has often been reported to produce false-positive results due to interference from background microorganisms or bioinformatic mismatches (Diao et al. 2023; Rausch et al. 2019). Overall, qPCR is considered a better approach than mNGS for the rapid and quantitative detection of known core microbes.

In this study, we developed a rapid method to detect and quantify 45 human gut core microbes. These microbes were selected as targets based on their high prevalence, abundance, and important ecological functions reported in previous metagenomic studies of human populations from multiple countries (Cheng et al. 2022; Gacesa et al. 2022; Liu et al. 2021; Olsson et al. 2022; Qin et al. 2010; Tramontano et al. 2018). We performed in silico analysis to obtain novel species-specific genetic markers and detection primers for 31 bacteria of the selected core microbes. A panel of SYBR Green–based qPCR assays was established to quantitatively detect the 45 core bacteria. The performance of the assays was comprehensively evaluated and further compared with mNGS by testing real fecal samples. Finally, we monitored and quantified the changes in abundance of the selected core microbes in 14 individuals over 8 weeks and constructed a visual bacterial profile to illustrate the stability of the core microbial community. By leveraging these qPCR assays, we hope to develop a novel appraisal system that can be used to rapidly assess the intestinal status and unscramble gut-related diseases.

## Materials and methods

### Bacterial culture and DNA extraction

A total of 64 bacterial strains were included in this study (Table S1 and S2). All bacteria were cultured under ATCC (American Type Culture Collection)– or DSMZ (Deutsche Sammlung von Mikroorganismen und Zellkulturen)–recommended conditions. Chromosomal DNA from the pure cultures was extracted using the QIAamp DNA Mini Kit (Cat. No. 51306; QIAGEN, Germany). The concentration and purity of the DNA were measured using the Thermo Scientific™ NanoDrop™ One instrument (Thermo Fisher Scientific, USA). The DNA concentration was greater than 10 ng/µL and the 260/280 nm absorbance ratio was between 1.8 and 2.0.

### Selection of species-specific genetic markers and primers for core microbes

Species-specific genetic markers and primers were selected in two steps. First, we conducted a comprehensive literature survey to search and verify previously reported specific primers that were used to detect these microbes (Ahmed et al. 2019; Chen et al. 2019; Gui et al. 2020; Laue et al. 2006; Li et al. 2020; Park et al. 2013; Schriefer et al. 2018; Takahashi et al. 2016; Tong et al. 2011; Yu et al. 2017; Yuli et al. 2005). Second, we performed the following in silico analysis for some bacteria with no available or qualified specific primers.

To construct local sequence databases for each target bacteria, we downloaded all genome sequence data (up to July 2021) from the National Center for Biotechnology Information (NCBI). We selected the representative strain sequence as the reference and constructed phylogenetic trees using the neighbor-joining method. Sequences with < 50% coverage were removed, and core genome sequence fragments were obtained by aligning the sequences and removing any gaps. Fragments with lengths of 100–600 bp were selected as candidates. We queried these candidates in the genome databases of other bacteria and removed the common parts. The remaining candidates were queried in the species-level local sequence databases, and fragments with low strain-level coverage were removed. In short, using local Basic Local Alignment Search Tool (BLAST) and coverage calculations, we removed the low-quality fragments. Finally, specific gene fragments of each target bacteria were obtained and used to design primers. The specificity of the selected gene fragments and primers was verified using online BLAST.

### Amplification and detection of pure DNA samples using qPCR

Amplification was performed using the Roche LightCycler® 480 System (Roche Diagnostics Ltd., Switzerland). The reaction was performed in a total volume of 20 µL, which comprised 10 µL of LightCycler® 480 SYBR® Green I Master (Roche Diagnostics GmbH, Germany), 6.4 µL of ultrapure water, 0.8 µL of each primer (10 µM), and 2 µL of DNA. The qPCR protocol consisted of four steps: preincubation at 95 °C for 5 min, amplification via 40 cycles of denaturation at 95 °C for 10 s and annealing at 60 °C for 1 min, melting curve analysis with the temperature continuously raised at a rate of 0.11 °C/s from 65 to 97 °C after 65 °C 1 min, and final cooling at 37 °C for 1 min. All primers were synthesized by Sangon Biotech (Shanghai, China). The cycle threshold (Ct) values were obtained using the LightCycler software (version 4.0) with the second derivative algorithms. The melting temperature (Tm) values were also obtained and used to verify the specificity of the amplification products. Samples with a typical amplification curve, Ct values less than those of the negative controls (ultrapure water), and Tm values consistent with the expected product were considered positive; otherwise, they were considered negative.

To determine the sensitivity of the qPCR assays, successive 10-fold dilutions of each target DNA (from 1 ng/µL to 0.1 pg/µL) were prepared and detected in triplicate. To validate the specificity, a DNA mixture sample that included the 45 core bacteria and 19 other intestinal bacteria (Table S2) was prepared with each bacterial DNA at a concentration of 1 ng/µL and detected using qPCR assays. The qPCR products (5 µL) were analyzed by performing 1.5% agarose gel electrophoresis at 100 V for 20 min, and the remaining samples were sent to Sangon Biotech (Shanghai, China) for Sanger sequencing.

### Subject criteria and fecal sample collection

All fecal samples were collected from healthy individuals. The inclusion criteria of individuals were as follows: (1) aged 25–35 years and not pregnant; (2) maintained consistent lifestyle and dietary habits over the preceding month; (3) no digestive disease, such as gastrointestinal tumors, polyps, IBD; (4) no other seriously bad health conditions, such as diabetes mellitus, cardiac disease, fatty liver disease, mental disorder; and (5) no adverse lifestyle habits like alcohol abuse. The exclusion criteria were as follows: (1) experienced short-term symptoms like diarrhea or fever within the last 2 weeks and (2) had used probiotics or antibiotics within the last month.

Fecal sample collection followed stringent protocols: immediately post-defecation, the middle uncontaminated portion of the feces was collected using a sterile spoon. Then, the fresh sample was promptly placed into a 50-mL sterile collector, which was labeled with identity information. The samples were placed on ice, transported to the laboratory within 1 h, and stored at −80 °C until DNA extraction was performed.

### Detection of fecal samples and analysis of species abundance by qPCR

Twenty-two fecal samples were collected from healthy individuals using the LONGSEEGEN Stool Storage Kit (Cat. No. LS-R-P-007; Guangdong Longsee, China). Genomic DNA was extracted using the SDS method (Lim et al. 2016) and diluted to 5 ng/µL. DNA samples were detected by qPCR as described in the “Amplification and detection of pure DNA samples by qPCR” section. The relative abundance of the 45 target bacteria in the sample was calculated using the 2^−ΔCt^ method (Gray et al. 2019), where the ΔCt value represented the difference between the Ct value of the target bacteria and the bacterial 16S rDNA gene ($\Delta\mathrm{Ct}=\mathrm{Ct}\;(\mathrm{target})-\mathrm{Ct}\;(16\mathrm S\;\mathrm{rDNA})$). A pair of universal primers located in the V6 region of the 16S rDNA gene was selected based on a previous study (Schriefer et al. 2018) and employed to quantify the bacterial 16S rDNA gene in samples.

### Metagenomic sequencing and relative abundance analysis

Genomic DNA from the 22 fecal samples was sequenced and analyzed. Sequencing libraries were generated using the NEBNext® Ultra™ DNA Library Prep Kit (NEB, USA) and sequenced on an Illumina HiSeq platform at Beijing Novogene Bioinformatics Technology Co., Ltd. Raw sequencing data were quality-controlled using Readfq (V8, https://github.com/cjfields/readfq) with the default settings. Human host–associated reads were identified using Bowtie 2.2.4 (Langmead and Salzberg 2012) and were removed before further analysis.

Filtered clean reads were assembled using MEGAHIT (Li et al. 2016) with the “presets meta-large” option. Assembled fragments over 500 bp were used for open reading frames (ORF) prediction by MetaGeneMark (http://topaz.gatech.edu/GeneMark/)*.* For predicted ORFs, CD-HIT (Fu et al. 2012) was adopted to remove redundancy and obtain the initial unique gene catalog (Unigenes). Clean reads were mapped to initial Unigenes using Bowtie 2.2.4 (Langmead and Salzberg 2012), and only genes with more than two mapped reads were kept in the final Unigenes. DIAMOND (Buchfink et al. 2015) was used to blast the final Unigenes to the sequences of bacteria, fungi, archaea, and viruses which were extracted from the nonredundant protein sequences database of NCBI. The lowest common ancestor algorithm in MEGAN (Gautam et al. 2023) was applied for taxonomic classification and relative abundance estimation of each microbe in the samples.

### Correlation analysis of the species abundance obtained by qPCR and mNGS

To compare the qPCR and mNGS methods’ ability to analyze the abundance of the 45 core microbes, we converted the results from each method into two corresponding matrices and flattened each matrix into 45 vectors. We then calculated the correlation between the resulting vectors and conducted a holistic comparison between the two methods using Pearson’s correlation coefficient analysis based on the space distances of the 45 vectors. Because the paired group data of species abundance resulting from qPCR and mNGS did not conform with normality, we compared each of the 45 core bacteria using the Spearman correlations analysis, respectively. The scale of the *x*/*y*-axis was transformed to log_10_. Statistical significance was set as follows: *, *P* < 0.05, **, *P* < 0.01, ***, *P* < 0.001, and ****, *P* < 0.0001.

### Dynamic detection of gut core microbes in individuals by qPCR

We collected fecal samples from another 14 individuals once a week for 2 months to observe changes in their core microbes over time. Any deviations from their normal lifestyle habits were recorded. All fecal samples were immediately stored at −80 °C after collection. After no more than 12 h of freezing, the samples were rapidly thawed at room temperature within 30 min and homogenized using sterile stir bars for 5 min. Fecal DNA was extracted using the QIAamp Power Fecal Pro DNA Kit (Cat. No. 51804; QIAGEN, Germany) following the manufacturer’s instructions and stored at −80 °C. The concentration and purity of the fecal DNA were evaluated using the Thermo Scientific™ NanoDrop™ One instrument (Thermo Fisher Scientific, USA). All fecal DNA samples were diluted to a concentration of 5 ng/µL for qPCR detection, as described in the “Amplification and detection of pure DNA samples by qPCR” section. The sum of the changes in the Ct values of each target bacteria ($\mathrm{Total}\;\Delta\mathrm{Ct}=\mathrm{Sum} \vert \Delta\mathrm{Ct}\;(\mathrm{weeks})-\Delta\mathrm{Ct}\;(\mathrm{first}\;\mathrm{week})\vert$) was used to represent the fluctuations in the overall gut core microbes over different weeks. The data were analyzed by one-way analysis of variance (ANOVA) and Tukey’s multiple comparisons test.

## Results

### Selection of gut core microbes and their molecular targets

**Fig. 1:**
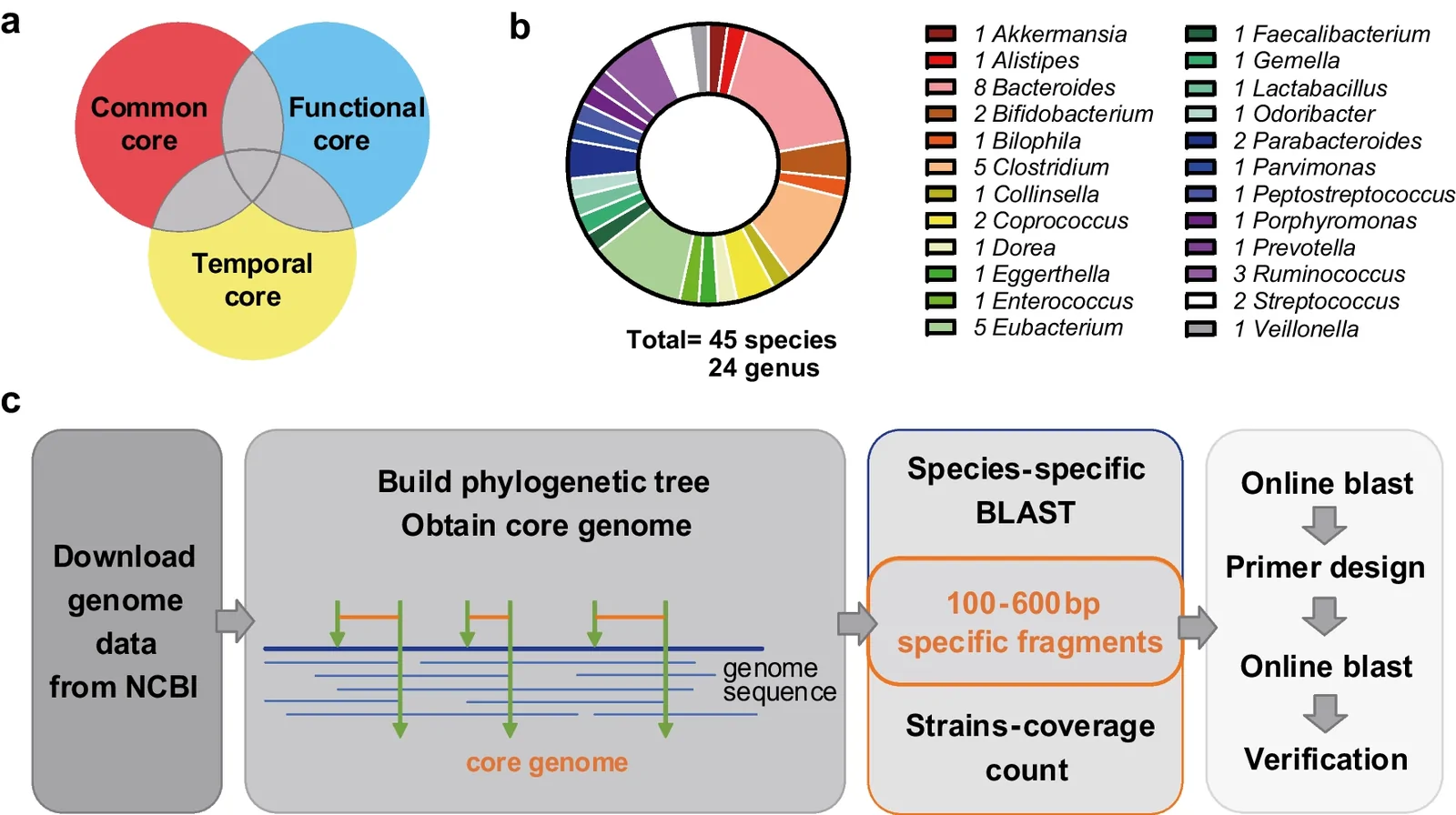
Selection of representative core microbes in the human gut and corresponding molecular targets. **a**, **b** Function and genus level distribution of the 45 selected bacteria. **c** Bioinformatic procedures for obtaining species-specific genetic markers and specific primers of the target bacteria

Based on previous metagenomic studies of gut microbiota in populations from multiple countries (Cheng et al. 2022; Gacesa et al. 2022; Liu et al. 2021; Olsson et al. 2022; Qin et al. 2010; Tramontano et al. 2018), a panel of 45 core bacteria from 24 genera was selected to serve as indicators to reflect the condition of the human gut core microbiota, including highly prevalent, abundant, and functional “driver” species in healthy individuals (Fig. 1, Table S1). All 45 core microbes had annotated genomes in the NCBI database. Among them, 14 bacteria had reported specific amplification primers, whereas the other 31 bacteria had no available or qualified primers. We performed in silico analysis to obtain novel species-specific genetic markers and primers for these 31 bacteria (Fig. 1c). The information regarding the gene targets and primer sequences used in the study are listed in Table 1.

**Table 1:** Information of the target bacteria, gene markers, and primers used in this study

Bacteria	Target gene	Sequences of the primers (5′-3′)	Size	Locus	References
*Akkermansia muciniphila*	*J4O16_01980*	F: TACAGCCCCGTTCACTCTCAT	113 bp	CP071807.1	This study
		R: TTAACGCTGACGGAGAATTGC			
*Alistipes shahii* ^*^	*AL1_17190*	F: CGCTACCTGACGCCTGAAG	100 bp	FP929032.1	This study
		R: GGCGTCCGGCATACTTGTA			
*Bacteroides caccae*	16S rDNA	F: AAACCCATACGCCGCAAG	69 bp		Tong et al. (2011)
		R: GACACCTCACGGCACGAG			
*Bacteroides dorei*	16S rDNA	F: ATCATGAGTTCACATGTCCG	126 bp		Ahmed et al. (2019)
		R: CTTCCTCTCAGAACCCCTATCC			
*Bacteroides eggerthii*	16S rDNA	F: CCCGATAGTATAGTTTTTCCGC	130 bp		Tong et al. (2011)
		R: TCCTCTCAGAACCCCTATCCAT			
*Bacteroides fragilis*	*BF9343_1564*	F: TGAGGCTGTTACGCGATTGA	126 bp	CR626927.1	This study
		R: GAACTAATGAGGAAAGCCATCGA			
*Bacteroides stercoris*	*rsfS*	F: CATAGCAGACCTTACCCGGATAGA	135 bp	CP081913.1	This study
		R: GGTTTGCTGTTCGCTCCTTT			
*Bacteroides thetaiotaomicron*	*FE838_17780*	F: GCGATGAGGCTGACAATGC	150 bp	CP040530.1	This study
		R: TGTAGACGGCCCGATGAAAG			
*Bacteroides uniformis*	16S rDNA	F: TCTTCCGCATGGTAGAACTATTA	146 bp		(Tong et al. (2011)
		R: ACCGTGTCTCAGTTCCAATGTG			
*Bacteroides vulgatus*	16S rDNA	F: CGGGCTTAAATTGCAGATGA	69 bp		(Tong et al. (2011)
		R: CATGCAGCACCTTCACAGAT			
*Bifidobacterium adolescentis*	*rnh*	F: GGATTCGGGTATGACCTTCGT	126 bp	AP009256.1	This study
		R: GCGGATTTCGGACAGACAAC			
*Bifidobacterium longum*	*yutF*	F: CGAGGAGTTGTTCGTGGTGTATT	111 bp	LR134369.1	This study
		R: CTCGACGGCGTGGTGTATC			
*Bilophila wadsworthia*	*taurine: pyruvate aminotransferase*	F: CCGATACCGACGCCTACTTC	128 bp		Laue et al. (2006)
		R: GCCCATCTTCGTGCAGTTTT			
*Clostridium bolteae*	16S rDNA	F: CCTCTTGACCGGCGTGT	157 bp		Yuli et al. (2005)
		R: CCTAGAGTGCCCAGCTTTACCTG			
*Clostridium butyricum*	*REGION: 3710956–3711084*	F: CTGTTCCAGGTGTTTCATAACTATCC	129 bp	CP040626.1	This study
		R: CATAGCATACAGCGAAGATTCAAGA			
*Clostridium leptum*	*CLOLEP_02038*	F: GCACTGGGTAGCGGTAATTTG	130 bp	DS480347.1	This study
		R: GCAGGCGGATAAGGAAAAGG			
*Clostridium ramosum* ^*^	*EYR00_13010*	F: GCTCATCATCGTCAATCACAAAG	127 bp	CP036346.1	This study
		R: GAGGAAATGTGCGGTTATTATGG			
*Clostridium saccharolyticum* ^*^	*Closa_3215*	F: TGCTTCCTCCACAATGGTCTCT	120 bp	CP002109.1	This study
		R: TCCTTTATGCCGGTGGCTAT			
*Collinsella aerofaciens*	*rsmH*	F: GAGGACCGTATCGTAAAGAACCA	138 bp	CP048433.1	This study
		R: ATCAGGCTGGGCAACCAA			
*Coprococcus catus* ^*^	*CC1_01340*	F: CTCAGGTTCGTTAGGATTTTTTGC	121 bp	FP929038.1	This study
		R: TGAACATACTTTGCTGCGGATT			
*Coprococcus comes* ^*^	*I6K69_16315*	F: CATTTTTTCGCCTTCCTCATTG	131 bp	CP070062.1	This study
		R: GAAGTATCTGGGAAGGACCGTTT			
*Dorea formicigenerans*	*DORFOR_00757*	F: CTGCACGGCTCCGATTCTAT	135 bp	AAXA02000010.1	This study
		R: TGGCAGGATTTCTTATGGAGAGA			
*Eggerthella lenta*	*Elen_2208*	F: GTTTCGTGTTCTGGCGTCAA	125 bp	CP001726.1	This study
		R: AGCGTCTGAACCGATTCTTTATTT			
*Enterococcus faecalis*	*nrdR*	F: CGTTATGCGTTGTCCAAGATGT	179 bp	CP008816.1	This study
		R: CACGATCCCCATTTTTCTTGA			
*Eubacterium eligens*	*EUBELI_01426*	F: AATCGCACCACACAGAATCAAT	149 bp	CP001104.1	This study
		R: GGGCAGAACACGCATTTTTC			
*Eubacterium hallii*	*EHLA_1509*	F: TGAAGGAAACAGAGCAGAAGGAT	151 bp	LT907978.1	This study
		R: ATGAGCCCTGTGAATCGCC			
*Eubacterium limosum*	*B2M23_19070*	F: CCAAAGGAAATCGCCATCAC	118 bp	CP019962.1	This study
		R: CGATGGTGGCTGGGTATTTT			
*Eubacterium rectale*	*MIO91_07280*	F: GCATAGTGTGTGAGTGCGGTAGA	141 bp	CP092643.1	This study
		R: CAGAAAGGCCTACGACAAGACA			
*Eubacterium siraeum* ^*^	*EUS_11480*	F: CCAAGGGCGAAAAGATCCA	143 bp	FP929044.1	This study
		R: AGCACTGCCTGCCTTGAAA			
*Faecalibacterum prausnitzii*	16S rDNA	F: GATGGCCTCGCGTCCGATTAG	198 bp		Gui et al. (2020)
		R: CCGAAGACCTTCTTCCTCC			
*Gemella morbillorum*	*NCTC11323_00317*	F: TGGCGCTCCATTATTTTGATATG	123 bp	LS483440.1	This study
		R: TCGCAAGAAGAGGTTCAAAGAAT			
*Lactobacillus ruminis*	*yihA*	F: GATTTCGTCTTGCGGGTTCA	115 bp	CP102284.1	This study
		R: TGATTTGCCGATTCTTGTCTGT			
*Odoribacter splanchnicus*	*LK432_02010*	F: AGTGGGCGATACAGGCATTC	186 bp	CP086000.1	This study
		R: TAGCCTCTTCCGACGACCAT			
*Parabacteroides distasonis*	*BDI_0504*	F: CGTGGTGACGCTGATTGG	186 bp	CP000140.1	This study
		R: TCCGTTTCCGTCAGCCTTAT			
*Parabacteroides merdae*	*INE87_01692*	F: GGCTCCCTTCCACCACATC	100 bp	CP072229.1	This study
		R: GCGTTCGGACAAAGTTGCA			
*Parvimonas micra*	*rpoB*	F: AAGAATGGAGAGAGTTGTTAGAGAAAGAA	133 bp		Yu et al. (2017)
		R: TTGTGATAATTGTGAAGAACCGAAGA			
*Peptostreptococcus anaerobius*	*REGION: 33704–33849*	F: CAAATAAAGTGTCATCTCCACCCTTAC	146 bp	KB906613.1	This study
		R: TGGTAGAGATTCTATATCAAAGAGACTTGG			
*Porphyromonas asaccharolytica*	*rpoB*	F: GAGCCAATCGTAAGGGAGGTAT	150 bp		Park et al. (2013)
		R: AGTCCGTTGCCGTGGTCTTGTG			
*Prevotella melaninogenica*	*rpoB*	F: TGACCGTAAGAAGAAGTTGCCTGTA	141 bp		Park et al. (2013)
		R: TTGCGACCGATAGCTGCCTTCATA			
*Ruminococcu bromii*	*rplC*	F: GTGCCTGCTCATCACCTTCA	147 bp	NPHY01000015.1	This study
		R: GACCCGGAATAGCACCCTTAA			
*Ruminococcu gnavus* ^*^	*rsxC*	F: GCACCGTTCGCAATGATG	100 bp	CP043051.1	This study
		R: AAGCCGGTGTTGTGGGAAT			
*Ruminococcu storques*	16S rDNA	F: GACGGTAATGCGTCCTTCC	129 bp		Takahashi et al. (2016)
		R: TGGCCGCTGGCTACTAAAG			
*Streptococcus parasanguinis*	*grpEL*	F: AGCAAGGAAGCCATTGCTCA	160 bp		Chen et al. (2019)
		R: GCATGCCTTCAACGACTTCC			
*Streptococcus salivarius*	*gtf*	F: CAACAGAGCGAGCAGAAGTTACTG	92 bp		Li et al. (2020)
		R: TACTGCTGCAGCTCTATCACTAGTTGT			
*Veillonella parvula*	*Vpar_1664*	F: ATTCCGCCCACTGTATTTGCT	128 bp	CP001820.1	This study
		R: ACGAGTGTTGGTGCATCTGG			
Universal 16S rDNA	*V6*	F: CAACGCGARGAACCTTACC	76 bp		Schriefer et al. (2018)
		R: ACAACACGAGCTGACGAC			

*The seven bacteria species with no reported specific detection primers

### Specificity, sensitivity, and quantitative features of the qPCR assays

**Fig. 2:**
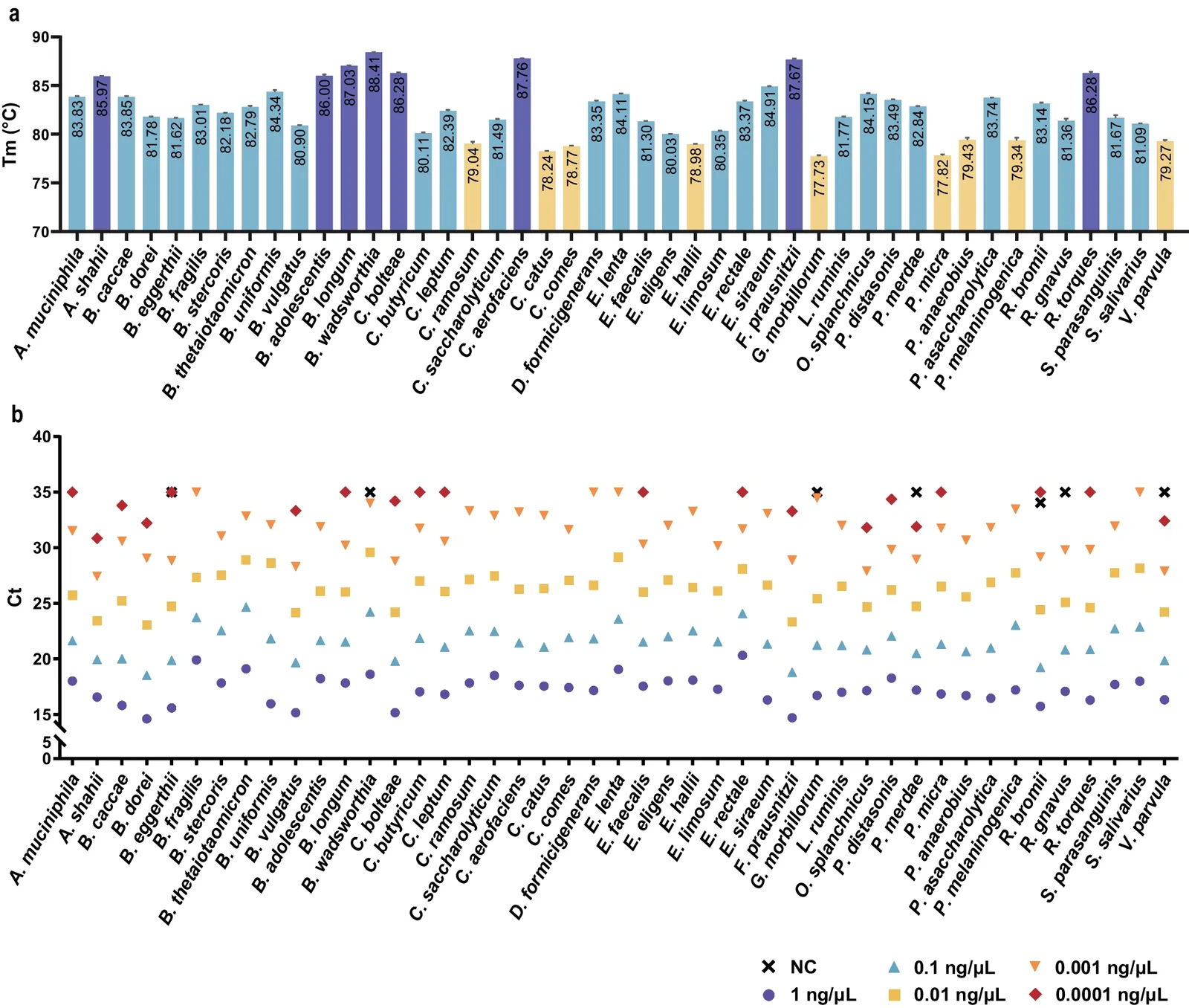
Performance of the qPCR assays for detection of the target bacteria. **a** Tm values of the amplification products of the qPCR assays. **b** Detection results (Ct values) of the target DNA dilutions with concentrations of 0.1 pg/µL to 1 ng/µL. NC refers to the negative control (ultrapure water)

A total of 45 SYBR Green−based qPCR assays were developed to detect the above core microbes. The amplification products ranged in length from 69 to 198 bp, with melting temperature (Tm) values ranging from 77.73 to 88.41 °C (Fig. 2a). The limit of detection (LOD) of the assays was determined to be between 0.1 and 1.0 pg/µL by testing pure bacterial genomic DNA samples (Fig. 2b, Table S3). The quantitative curve was established for each target with log_10_ (the DNA concentration, ng/µL) as the *x*-axis and the Ct value as the *y*-axis. These curves demonstrated good linearity for the targets at concentrations ranging from 0.1 pg/µL to 1.0 ng/µL or from 1.0 pg/µL to 1.0 ng/µL, with the coefficient of determination (*R*^2^) ranging from 0.9669 to 0.9994 (depending on the targets, details in Table S3).

To evaluate the specificity of the qPCR assays, DNA mixture samples containing all 45 target bacteria and 19 other common gut bacteria (Table S2) were prepared and tested. The size and sequence data of the amplified products were consistent with the results of the pure target DNA samples, as demonstrated in agarose gel electrophoresis and Sanger sequence analysis, demonstrating that the designed primers and assays were highly specific to the corresponding targets.

### Correlation analysis of qPCR and mNGS for analyzing real fecal samples

A total of 22 fecal samples from healthy individuals were collected and tested using both the qPCR and mNGS methods. For the qPCR method, we first estimated the overall bacterial abundance through amplification and quantification of the bacterial 16S rDNA gene with universal primers located in the V6 region. Then, we determined the relative abundance of each core bacteria in the sample. Overall, the results showed high consistency (Pearson’s *r* = 0.8688, *P* < 0.0001) between the two methods in terms of quantifying the species abundance of the 45 bacteria in the real samples, which demonstrated considerable competitiveness of the qPCR method for quantifying known core microbes.

**Fig. 3:**
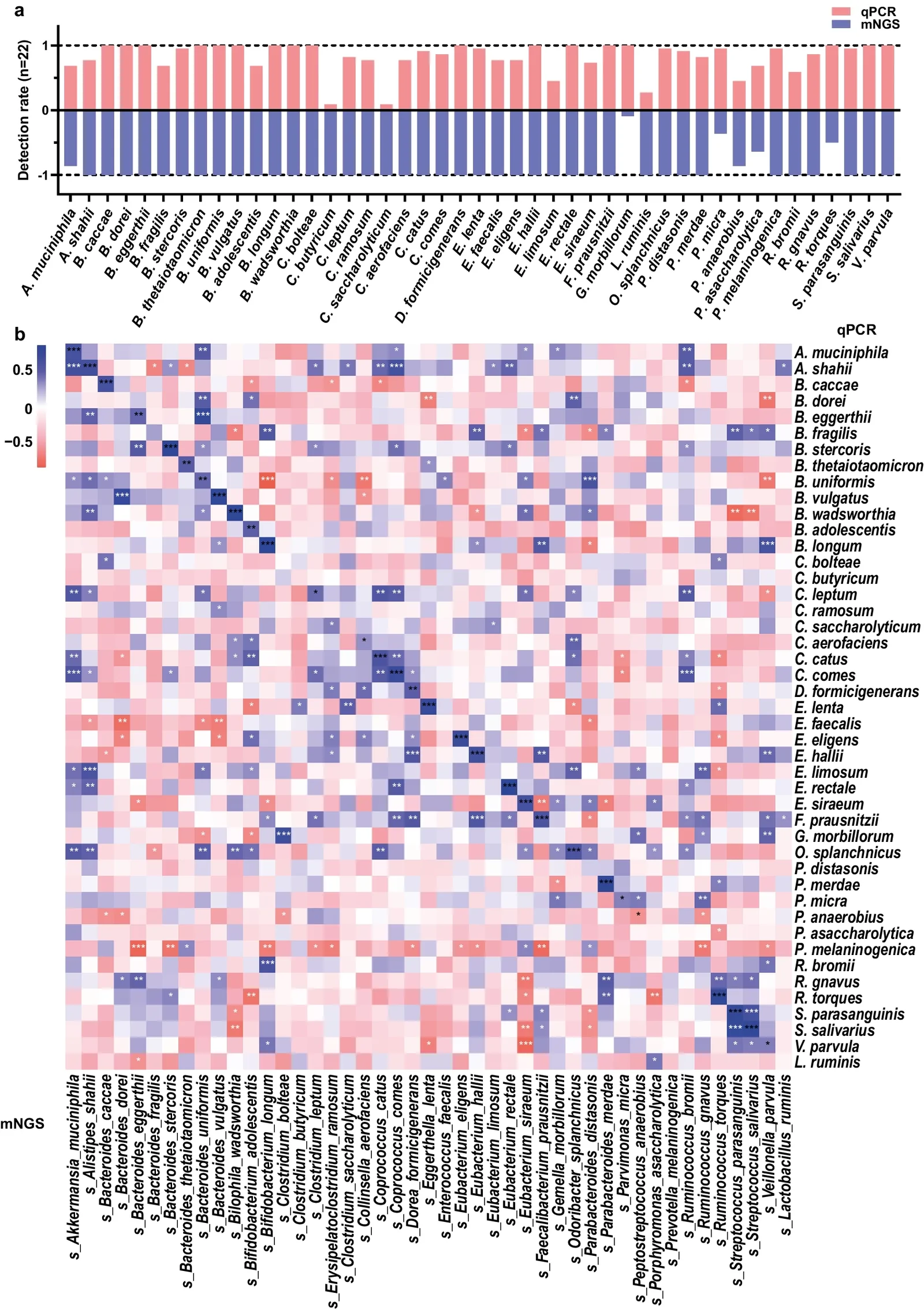
Correlation analysis of qPCR and mNGS in the analysis of 22 fecal samples. **a** The detection rates of the 45 core microbes obtained by qPCR and mNGS. **b** Spearman’s correlation analysis of the species abundance obtained by qPCR and mNGS. *, *P* < 0.05, **, *P* < 0.01, and ***, *P* < 0.001

By the qPCR method, 35 of the 45 core bacteria were detected with a high detection rate of over 70% (16/22), whereas the detection rate of 5 bacteria was between 50% (11/22) and 70% and that of other 5 bacteria were below 50%, including *Clostridium butyricum* (9%, 2/22), *Clostridium saccharolyticum* (9%, 2/22), *Lactobacillus ruminis* (27%, 6/22), *Eubacterium limosum* (45%, 10/22), and *Peptostreptococcus anaerobius* (45%, 10/22) (Fig. 3a). The mNGS method had a high detection rate (100%) for 39 target bacteria. However, its detection rates for other three bacteria were no more than 50%, including *Gemella morbillorum* (9%, 2/22), *Parvimonas micra* (36%, 8/22), and *Ruminococcus torques* (50%, 11/22). Overall, in 22 fecal samples, approached or exceeded 70% detection rates were achieved for 41 core bacteria by mNGS, and for 39 core bacteria by PCR. Albeit with a small sample size, this result suggests the universality of these core microbes in the human gut, as observed in the metagenomic data from multiple countries in previous studies (Olsson et al. 2022; Tramontano et al. 2018).

The abundance analysis of each target bacteria in the samples revealed that the abundance of 29 of the 45 bacteria was significantly correlated between the two methods, whereas that of the remaining 16 was not correlated or negatively correlated (Fig. 3b, Fig. S1), including all 5 bacteria with a qPCR detection rate below 50%. Although both methods had a high detection rate of ≥ 90% for some targets, such as *Bacteroides dorei*, *Clostridium bolteae*, *Parabacteroides distasonis*, and *Prevotella melanogenic*, their abundances were not correlated between the two methods. This was probably due to that the accuracy of mNGS is affected by substantial interference from background microorganisms, usually resulting in false-positive results. Meanwhile, qPCR may also produce some false-negative results for certain bacteria, because of the insufficient conservativeness of the selected gene targets or low sensitivity, which still needs more optimizations.

### Dynamic profile of human gut core microbes by qPCR

**Fig. 4:**
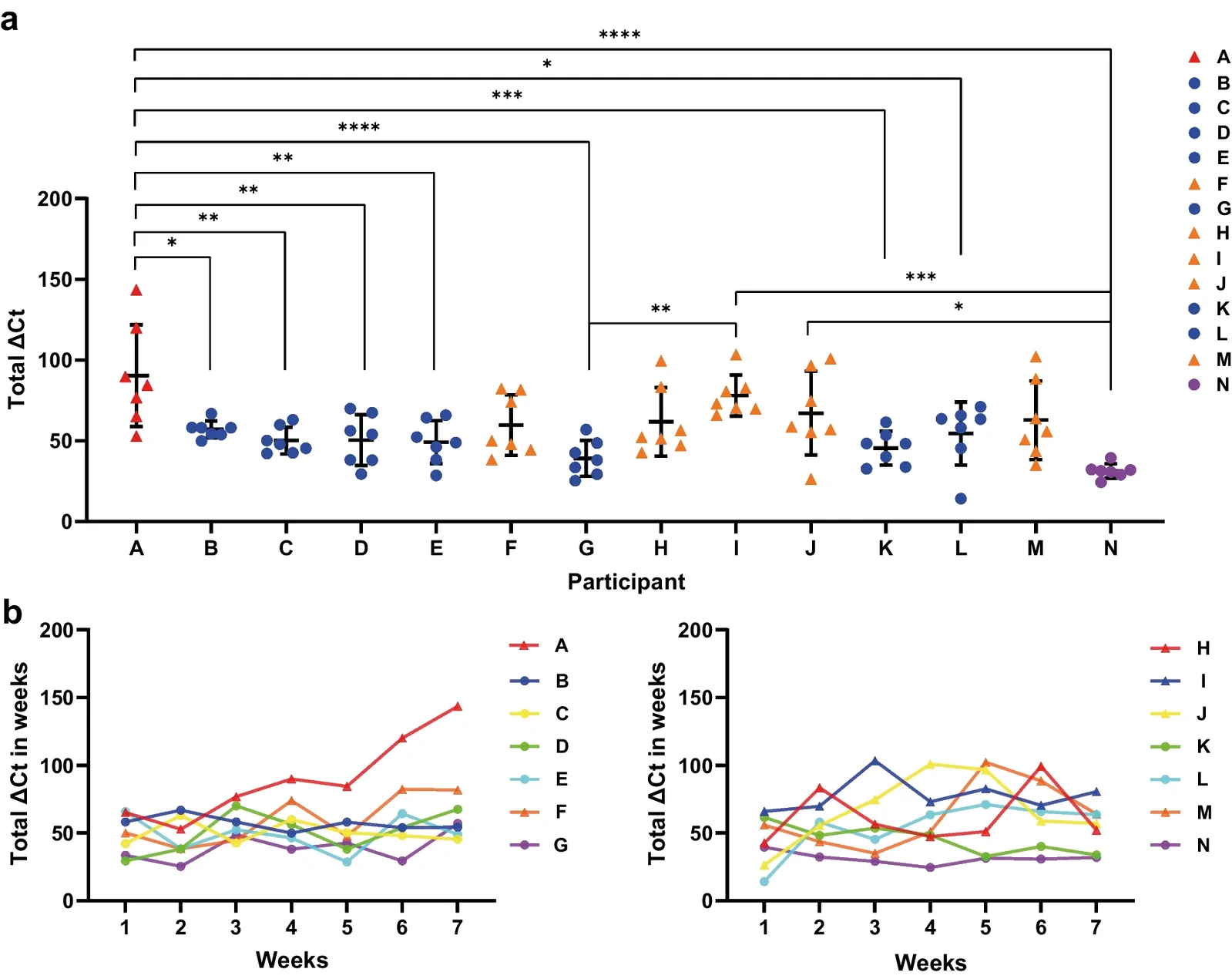
Dynamic changes in species abundance of the gut core microbes in 14 individuals over 8 weeks. **a** The sum of the changes in Ct values of all target bacteria (Total ΔCt) (*y*-axis) was used to represent the fluctuation of core microbes for individuals (*x*-axis), with the first week’s data as the baseline. The data were analyzed by one-way ANOVA. *, *P* < 0.05, **, *P* < 0.01, ***, *P* < 0.001 and ****, *P* < 0.0001. **b** Dynamic changes of the gut core microbes over 8 weeks for each individual, with total ΔCt values as the *y*-axis and time (weeks) as the *x*-axis

A total of 112 samples were collected from 14 healthy individuals (once a week for 8 weeks) and analyzed by the developed qPCR assays. The results of the 29 core microbes, whose abundance was significantly correlated between the two methods, were used to analyze dynamic changes in the gut microbiota of the individuals. As shown in Fig. 4, most participants had relatively stable core microbe profiles over the 8 weeks, except for participant A, who exhibited significant fluctuations in the profile. This individual suffered a brief bout of fever and diarrhea and took antipyretic and antidiarrheal drugs during the second week; the total abundance changes of the core microbes (total ΔCt) began to increase over the next few weeks, especially in weeks 6 (total ΔCt = 120.03) and 7 (total ΔCt = 143.52) (Fig. 4b). This result implies that it may take a certain amount of time for individuals to repair or rebuild their gut microbiota after exposure to strong perturbations, which requires more detailed research.

**Fig. 5:**
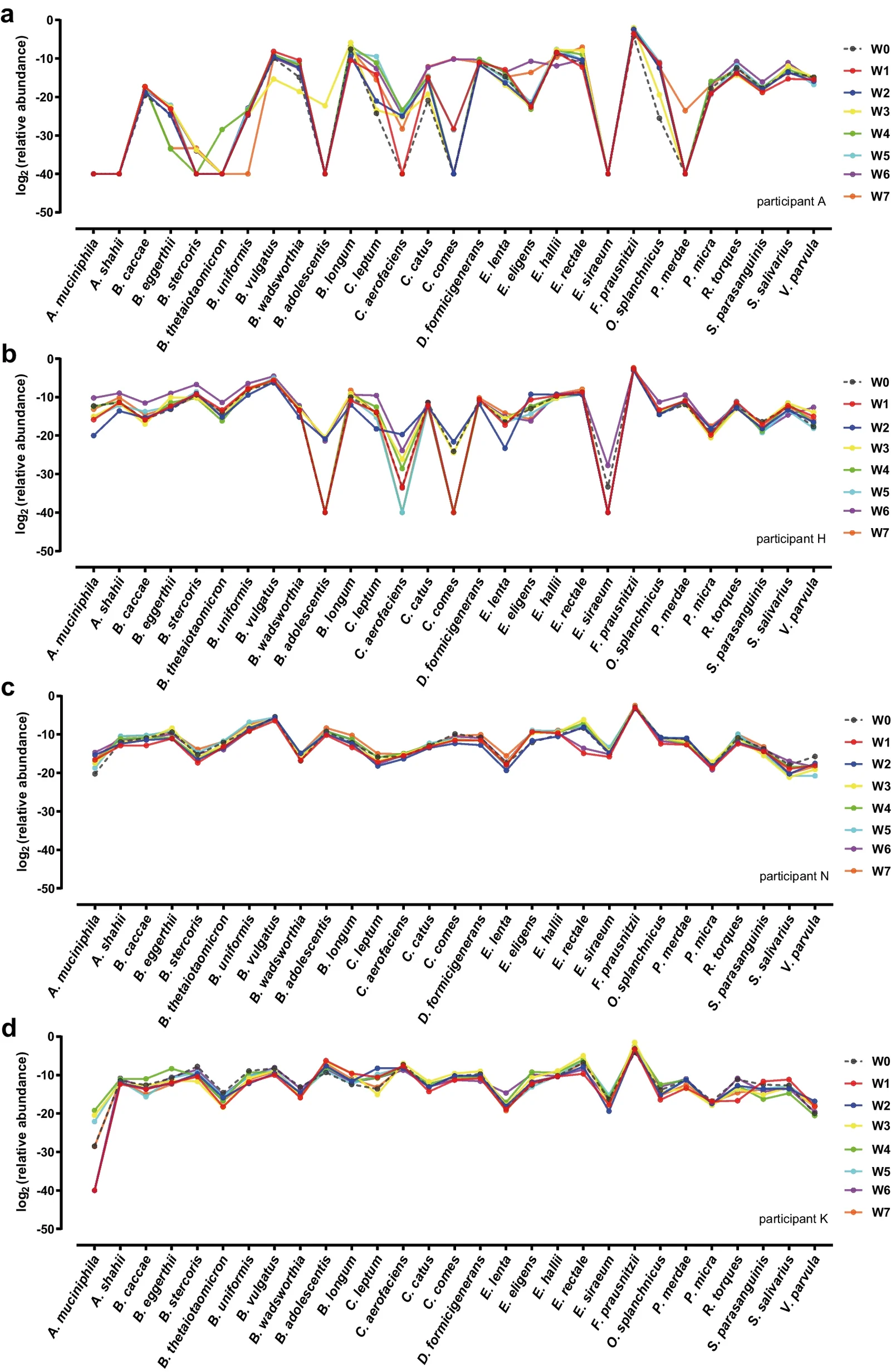
Dynamic profiles of gut core microbes obtained from the results of participants A, H, N, and K. The *x*-axis and *y*-axis represent the 29 selected core microbes and the abundance [log_2_ (relative abundance)] of each target, respectively

Slight fluctuations (total ΔCt ≤ 103.45) occurred in five individuals (participants F, H, I, J, and M), although they maintained the same lifestyle habits, did not become ill, and took no medications during the study period. In addition, participant N continuously took ibuprofen capsules and diclofenac for 4 weeks during the study period to treat temporomandibular joint osteoarthritis (without fever); however, the core microbes remained relatively stable (total ΔCt ≤ 39.51). For the other participants (total ΔCt ≤ 71.12), such as participant K, the abundance of core microbes was highly stable over the 8 weeks. The dynamic changes in the species abundance of each target bacteria for the 14 participants are described in Fig. 5 and Fig. S2. Overall, the gut core microbes of most participants are relatively stable or have certain fluctuations under a constant environment, which has the potential to be employed as an indicator of human health or disease status.

## Discussion

In this study, we developed a panel of species-specific qPCR-based assays for the rapid detection of 45 core microbes in the human gut. Through a bioinformatic pipeline, a total of 31 sets of specific fragments and primers were newly identified to detect those core bacteria with no available or qualified specific primers. After comprehensive evaluation, our assays presented a high specificity and sensitivity (0.1 to 1.0 pg/µL) to these 45 core microbes, as well as a high consistency with mNGS in quantifying core microbes in real fecal samples. Further, this panel was used to quantify the dynamics of core microbes in 14 individuals for 8 weeks, showing considerable stability of the core microbes for most participants. Our study exhibited great potential of this panel for rapid profiling and regular monitoring of gut core microbiota in individuals. In terms of contributing to clinical application, such as fecal microbiota transplantation, our approach may be used for the convenient and cost-effective detection of healthy-related core microbes in donor samples and the evaluation of structural reconstitution of core microbes in patients after treatment.

Providing insights into what constitutes healthy microbiota is one of the important clinical goals in human gut microbiome research (Shanahan et al. 2021). Current studies about the gut microbiome primarily focus on various diseases or unhealthy conditions to explore the impact of gut microbiota in single time point–based cross-sectional studies. However, the dynamic of gut microbes in healthy individuals is easily overlooked but equally important. In this study, we focused on the core microbes related to human health and dynamically quantified 29 of them from healthy individuals for 8 weeks using the developed qPCR assays. The core microbe profiles of most participants showed notable stability or minor fluctuations. This result ties well with a previous longitudinal 1-year population study (Olsson et al. 2022). In addition, the colonization of these core members (rooting in *Prevotella*, *Bacteroides*, and *Faecalibacterium*) with high abundance is more resistant to species loss contributing to community stability (Revel-Muroz et al. 2023). Reduction and even depletion of some core microbes, such as *Faecalibacterium prausnitzii* and *Eubacterium hallii* (butyric acid producer), have been observed in patients with IBD (Ning et al. 2023). These findings further highlight the indispensable effect of core microbes on the stability of the human gut microbiome. Paying more attention to the dynamic oscillations of core microbes and their normal baseline is necessary to expand our understanding of health status.

Another noteworthy point is the choice of measurement technique to develop a rapid and simple appraisal system for profiling the human gut core microbiota in practice. In the study, we tested 22 real fecal samples by two common measurement techniques, qPCR and mNGS. The two methods showed high consistency in quantifying the abundance of the selected core microbes in human fecal samples, suggesting the potential of qPCR for rapidly profiling species-level signatures of the core microbiota to some extent. The qPCR-based study is targeted and requires knowledge of the microbial targets in advance. Generally, it is used to rapidly detect and quantify the target of interest in a large sample at low cost (Sung et al. 2023). mNGS is a non-targeted tool for quantifying gut microbiota signatures with species- or strain-level taxonomic resolution. Currently, some disease-specific gut microbiota signatures grow clear in mNGS-based studies (Duvallet et al. 2017; Zhang et al. 2022). Some diagnostic models at the species level have been developed based on metagenomic datasets and have shown good performance in cohort validations (Ning et al. 2023; Oh et al. 2020). Given the above, the combination of mNGS and qPCR is hopeful to translate these microbial signatures from massive metagenomic data into practical clinical applications for health monitoring, disease diagnosis, and treatment strategy.

Although the two methods showed high consistency in quantifying core microbes, some inconsistent results were observed in the detection of *C. butyricum*, *C. saccharolyticum*, *L. ruminis*, *E. limosum*, *P. anaerobius*, and *R. bromii*. Their detection rates were low by qPCR but high by mNGS. On one hand, mNGS may easily generate false-positive results, especially for low-abundance bacteria in samples. This is due to bioinformatic classification mismatches or interference from background microorganisms or taxonomically close-related species (Lindstedt et al. 2022). On the other hand, the gene fragments and primers we obtained may be insufficiently conserved to detect all strains of the target bacteria. This issue may be particularly relevant for certain microbes whose annotated genomes are few in the NCBI database, such as *C. saccharolyticum* (only 4 genome sequences previously). With such a small number of sequences, it is difficult to guarantee that the designed primers have ideal coverage at the strain level (Table S3). Under this condition, qPCR may also produce false-negative results. Therefore, it is necessary to enrich more annotated sequences of related gut microbes to facilitate the discovery of highly conserved and specific genetic markers, thereby improving the accuracy of mNGS and qPCR methods.

There are several limitations in the study. First, the current system contains a subset of the core microbiota; however, there are still many prevalent or functionally important microbes that need to be investigated, such as members of the *Prevotella*, *Roseburia, Dorea*, and *Lactobacillus* genera (Nie et al. 2021; Tett et al. 2021; Xiao et al. 2020). Therefore, the detection list needs to be continuously enriched to establish a more extensive appraisal system for assessing the gut core microbiota. In addition, constantly optimizing the detection primers is necessary as more and more genome sequences of gut bacteria are published and shared, especially for bacteria with fewer published annotated genome sequences previously. Third, the current qPCR assays employ single-target detection, which is far from the requirements of clinic applications and other large-scale investigations. Multiple-detection strategies are recommended to improve the efficiency of our assays, such as the TaqMan Array Card (Liu et al. 2013), SAMBA (Luo et al. 2022), and MeltArray (Huang et al. 2022).

In conclusion, this study established a panel of qPCR assays for the rapid and quantitative detection of core microbes in the human gut. This panel can be applied for the rapid profiling and regular monitoring of gut core microbiota in individuals, and may further help to assess the intestinal status and unscrambling gut-related diseases. Further research is warranted to improve the system, and a considerable sample size is required to support the investigation of gut core microbiota in populations and its clinical value. Nevertheless, our study provides a fundamental basis for developing more efficient ways to understand the gut core microbiota.

## Electronic supplementary material

Below is the link to the electronic supplementary material.

Supplementary Material 1

## Data Availability

All sequencing data used in this study have been deposited in the Genome Sequence Archive in National Genomics Data Center, China National Center for Bioinformation/Beijing Institute of Genomics, Chinese Academy of Sciences (GSA: CRA011952) that are publicly accessible at https://ngdc.cncb.ac.cn/gsa.
