# Supplementary material for: Quantitative and dynamic profiling of human gut core microbiota by real-time PCR
Source: Appl Microbiol Biotechnol. 2024 Jun 26;108:396. doi: 10.1007/s00253-024-13204-4 (PMC11208268; doi:10.1007/s00253-024-13204-4)
Supplement: Supplementary file 1 [file 253_2024_13204_MOESM1_ESM.pdf]

Supplementary Material

Quantitative and Dynamic Profiling of Human Gut Core Microbiota by Real-time PCR

Ziheng Yan[a], Tongyu Hao[a], Yanfeng Yan[a], Yanting Zhao[a], Yarong Wu[a], Yafang Tan[a], Yujing Bi[a], Yujun Cui[a], Ruifu Yang[a, b], Yong Zhao[a, b],

[a] State Key Laboratory of Pathogen and Biosecurity, Beijing Institute of Microbiology and Epidemiology, 100071 Beijing, China

[b] Beijing Key Laboratory of POCT for Bioemergency and Clinic, 100071 Beijing, China

Address correspondence to Yong Zhao, zhaoyong179@139.com; Ruifu Yang, ruifuyang@gmail.com

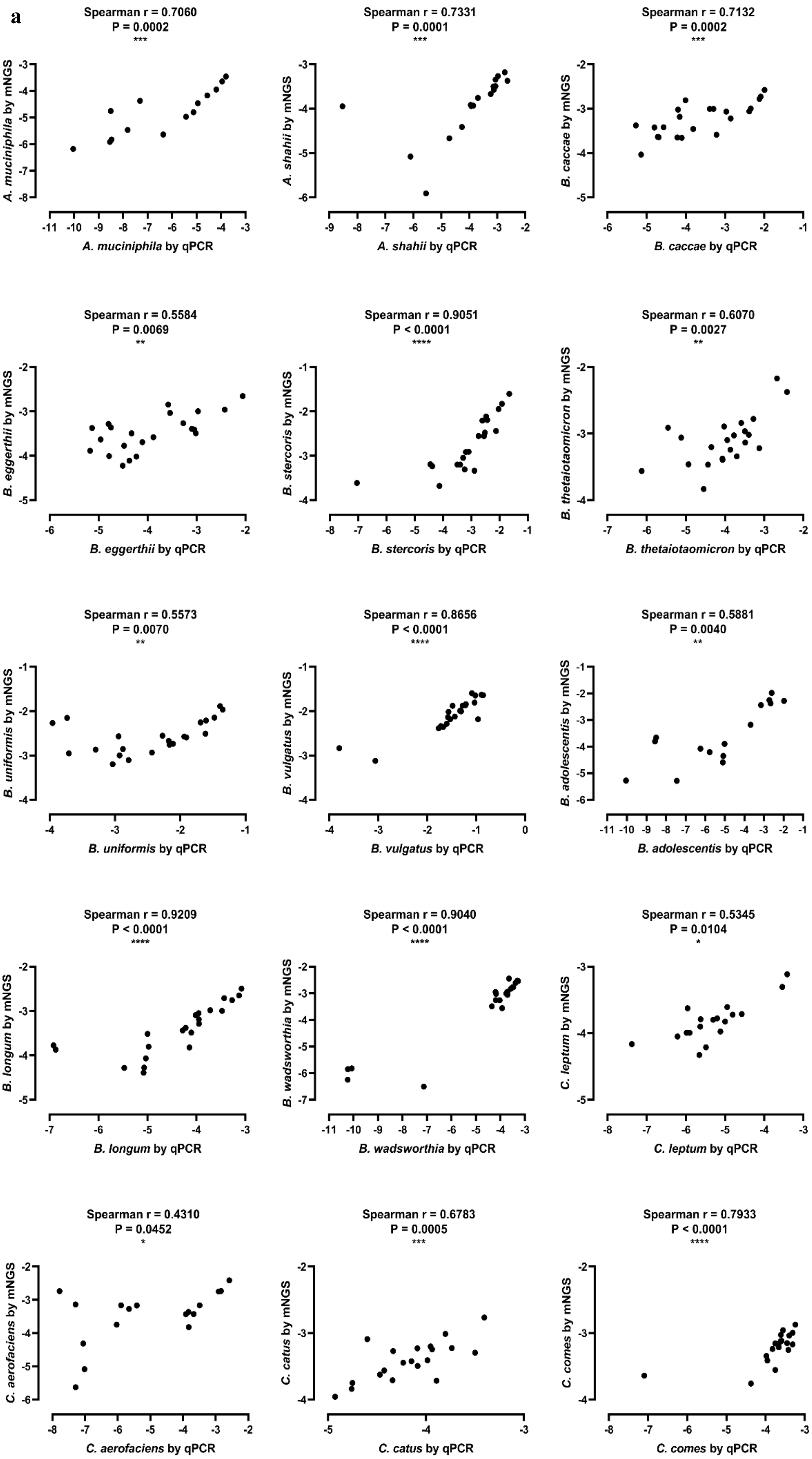
a
Spearman r = 0.7060
P = 0.0002
***
A. muciniphila by mNGS
A. muciniphila by qPCR
Spearman r = 0.7331
P = 0.0001
***
A. shahii by mNGS
A. shahii by qPCR
Spearman r = 0.7132
P = 0.0002
***
B. caccae by mNGS
B. caccae by qPCR
Spearman r = 0.5584
P = 0.0069
**
B. eggerthii by mNGS
B. eggerthii by qPCR
Spearman r = 0.9051
P < 0.0001
****
B. stercoris by mNGS
B. stercoris by qPCR
Spearman r = 0.6070
P = 0.0027
**
B. thetaiotaomicron by mNGS
B. thetaiotaomicron by qPCR
Spearman r = 0.5573
P = 0.0070
**
B. uniformis by mNGS
B. uniformis by qPCR
Spearman r = 0.8656
P < 0.0001
****
B. vulgatus by mNGS
B. vulgatus by qPCR
Spearman r = 0.5881
P = 0.0040
**
B. adolescentis by mNGS
B. adolescentis by qPCR
Spearman r = 0.9209
P < 0.0001
****
B. longum by mNGS
B. longum by qPCR
Spearman r = 0.9040
P < 0.0001
****
B. wadsworthia by mNGS
B. wadsworthia by qPCR
Spearman r = 0.5345
P = 0.0104
*
C. leptum by mNGS
C. leptum by qPCR
Spearman r = 0.4310
P = 0.0452
*
C. aerofaciens by mNGS
C. aerofaciens by qPCR
Spearman r = 0.6783
P = 0.0005
***
C. catus by mNGS
C. catus by qPCR
Spearman r = 0.7933
P < 0.0001
****
C. comes by mNGS
C. comes by qPCR

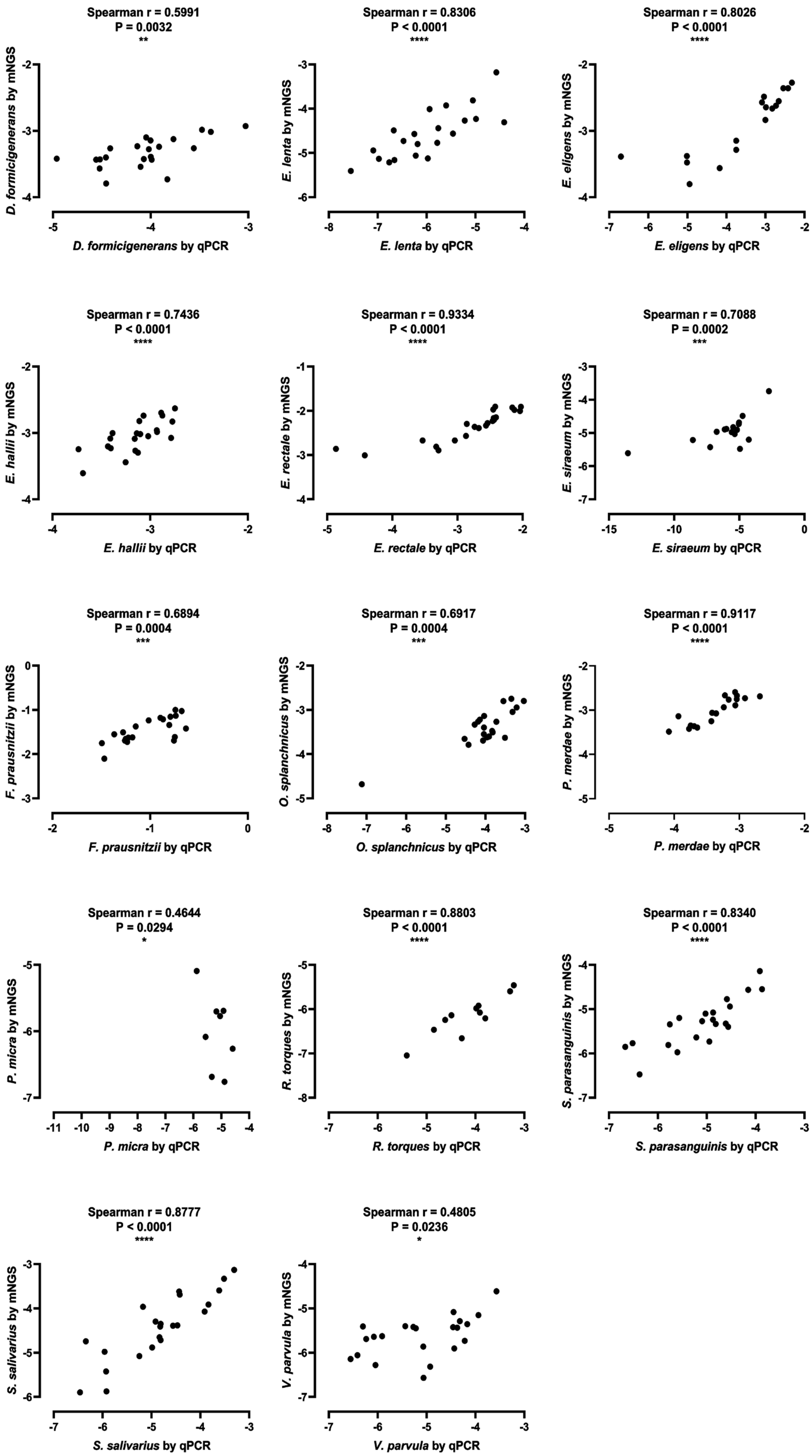
Spearman r = 0.5991
P = 0.0032
**
D. formicigenerans by mNGS
D. formicigenerans by qPCR
Spearman r = 0.8306
P < 0.0001
****
E. lenta by mNGS
E. lenta by qPCR
Spearman r = 0.8026
P < 0.0001
****
E. eligens by mNGS
E. eligens by qPCR
Spearman r = 0.7436
P < 0.0001
****
E. hallii by mNGS
E. hallii by qPCR
Spearman r = 0.9334
P < 0.0001
****
E. rectale by mNGS
E. rectale by qPCR
Spearman r = 0.7088
P = 0.0002
***
E. siraeum by mNGS
E. siraeum by qPCR
Spearman r = 0.6894
P = 0.0004
***
F. prausnitzii by mNGS
F. prausnitzii by qPCR
Spearman r = 0.6917
P = 0.0004
***
O. splanchnicus by mNGS
O. splanchnicus by qPCR
Spearman r = 0.9117
P < 0.0001
****
P. merdae by mNGS
P. merdae by qPCR
Spearman r = 0.4644
P = 0.0294
*
P. micra by mNGS
P. micra by qPCR
Spearman r = 0.8803
P < 0.0001
****
R. torques by mNGS
R. torques by qPCR
Spearman r = 0.8340
P < 0.0001
****
S. parasanguinis by mNGS
S. parasanguinis by qPCR
Spearman r = 0.8777
P < 0.0001
****
S. salivarius by mNGS
S. salivarius by qPCR
Spearman r = 0.4805
P = 0.0236
*
V. parvula by mNGS
V. parvula by qPCR

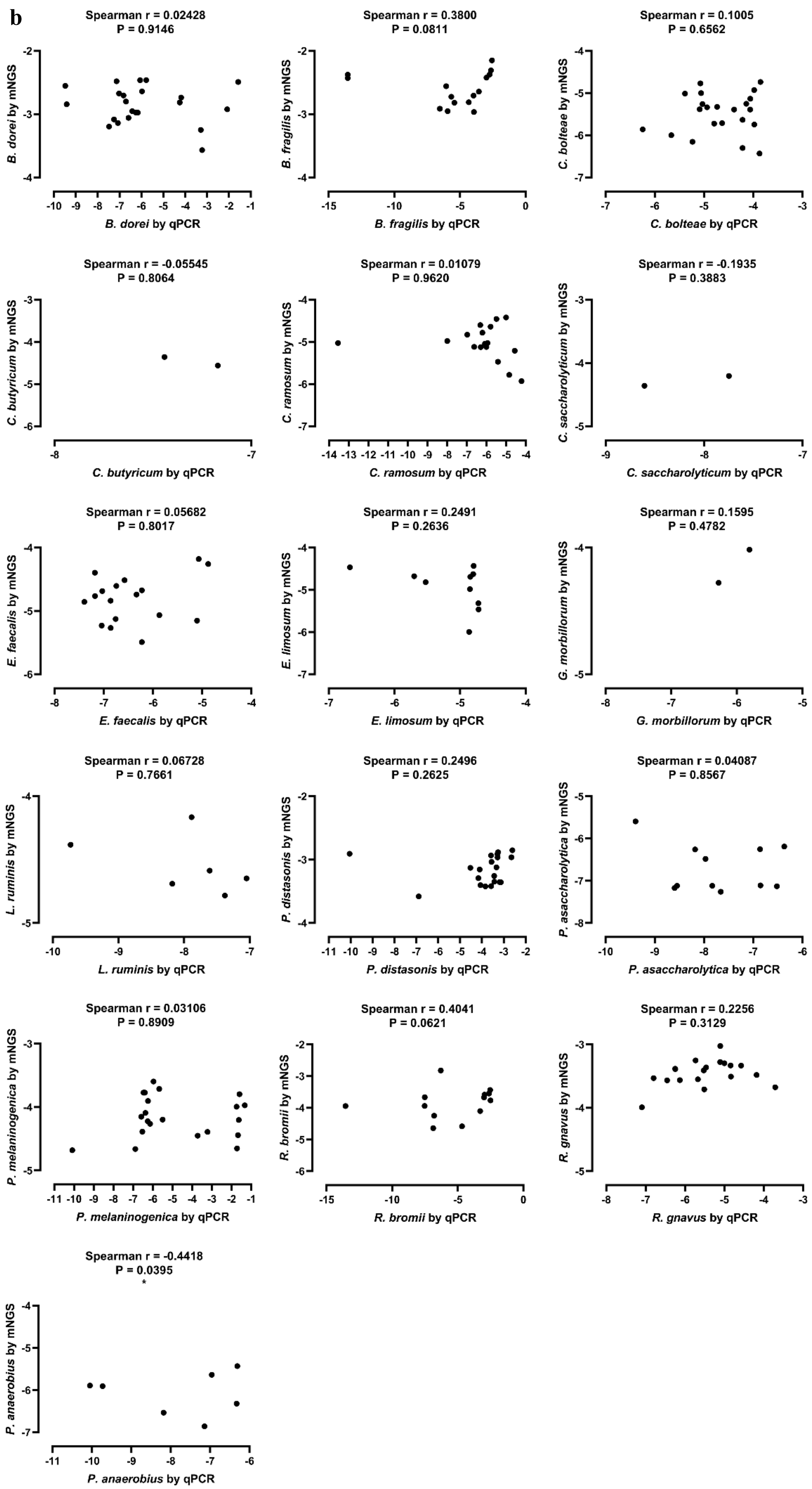
b
Spearman r = 0.02428
P = 0.9146
B. dorei by mNGS
B. dorei by qPCR
Spearman r = 0.3800
P = 0.0811
B. fragilis by mNGS
B. fragilis by qPCR
Spearman r = 0.1005
P = 0.6562
C. bolteae by mNGS
C. bolteae by qPCR
Spearman r = -0.05545
P = 0.8064
C. butyricum by mNGS
C. butyricum by qPCR
Spearman r = 0.01079
P = 0.9620
C. ramosum by mNGS
C. ramosum by qPCR
Spearman r = -0.1935
P = 0.3883
C. saccharolyticum by mNGS
C. saccharolyticum by qPCR
Spearman r = 0.05682
P = 0.8017
E. faecalis by mNGS
E. faecalis by qPCR
Spearman r = 0.2491
P = 0.2636
E. limosum by mNGS
E. limosum by qPCR
Spearman r = 0.1595
P = 0.4782
G. morbillorum by mNGS
G. morbillorum by qPCR
Spearman r = 0.06728
P = 0.7661
L. ruminis by mNGS
L. ruminis by qPCR
Spearman r = 0.2496
P = 0.2625
P. distasonis by mNGS
P. distasonis by qPCR
Spearman r = 0.04087
P = 0.8567
P. asaccharolytica by mNGS
P. asaccharolytica by qPCR
Spearman r = 0.03106
P = 0.8909
P. melaninogenica by mNGS
P. melaninogenica by qPCR
Spearman r = 0.4041
P = 0.0621
R. bromii by mNGS
R. bromii by qPCR
Spearman r = 0.2256
P = 0.3129
R. gnavus by mNGS
R. gnavus by qPCR
Spearman r = -0.4418
P = 0.0395
*
P. anaerobius by mNGS
P. anaerobius by qPCR

Fig. S1 Correlation analysis of the relative abundance of 45 bacteria determined by qPCR and mNGS. **a** The species abundances of 29 bacteria obtained by the two methods were significantly correlated ($P < 0.05$); **b** the other 16 bacteria were not correlated ($P > 0.05$). The data were analyzed by Spearman's correlation (n=22). Since the scale of the x/y axis was converted to $\log_{10}$, the results of undetected targets are not included in the graphs

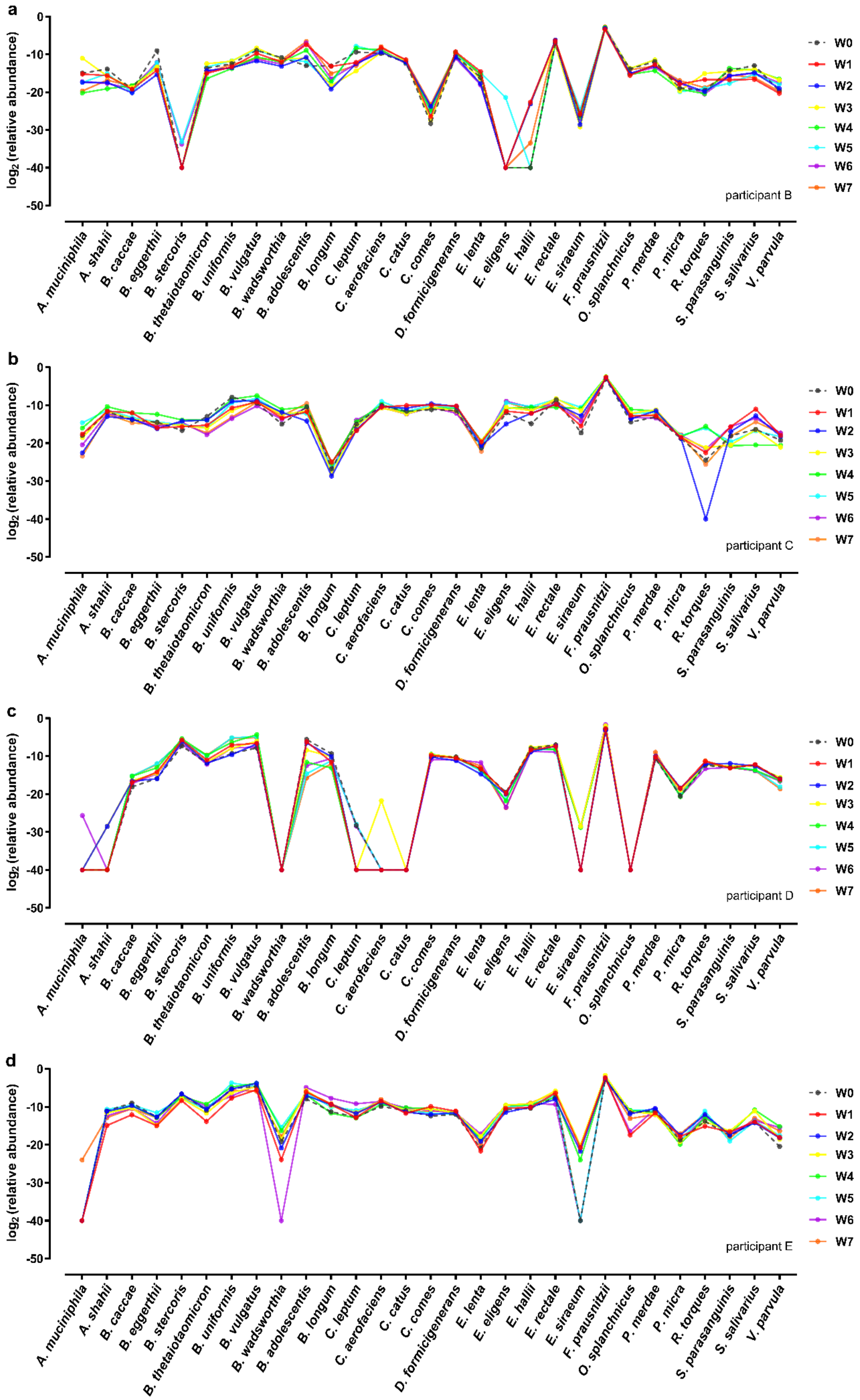
a
b
c
d
log2 (relative abundance)
participant B
participant C
participant D
participant E
W0
W1
W2
W3
W4
W5
W6
W7
A. muciniphila
A. shahii
B. caccae
B. eggerthii
B. stercoris
B. thetaiotaomicron
B. uniformis
B. vulgatus
B. wadsworthia
B. adolescentis
B. longum
C. leptum
C. aerofaciens
C. catus
C. comes
D. formicigenerans
E. lenta
E. eligens
E. hallii
E. rectale
E. siraeum
F. prausnitzii
O. splanchnicus
P. merdae
P. micra
R. torques
S. parasanguinis
S. salivarius
V. parvula

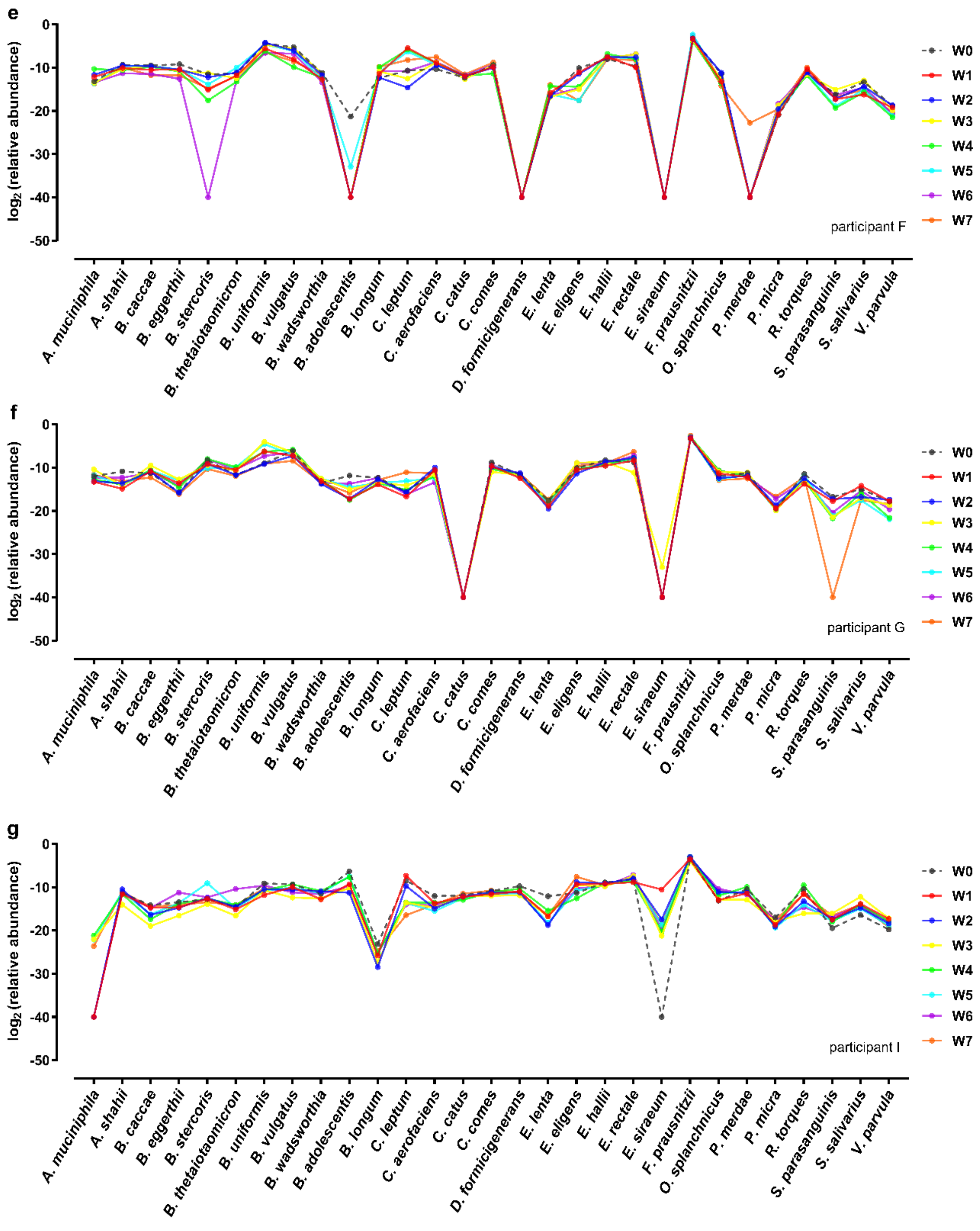
e
f
g
log2 (relative abundance)
0
-10
-20
-30
-40
-50
participant F
participant G
participant I
W0
W1
W2
W3
W4
W5
W6
W7
A. muciniphila
A. shahii
B. caccae
B. eggerthii
B. stercoris
B. thetaiotaomicron
B. uniformis
B. vulgatus
B. wadsworthia
B. adolescentis
B. longum
C. leptum
C. aerofaciens
C. catus
C. comes
D. formicigenerans
E. lenta
E. eligens
E. hallii
E. rectale
E. siraeum
F. prausnitzii
O. splanchnicus
P. merdae
P. micra
R. torques
S. parasanguinis
S. salivarius
V. parvula

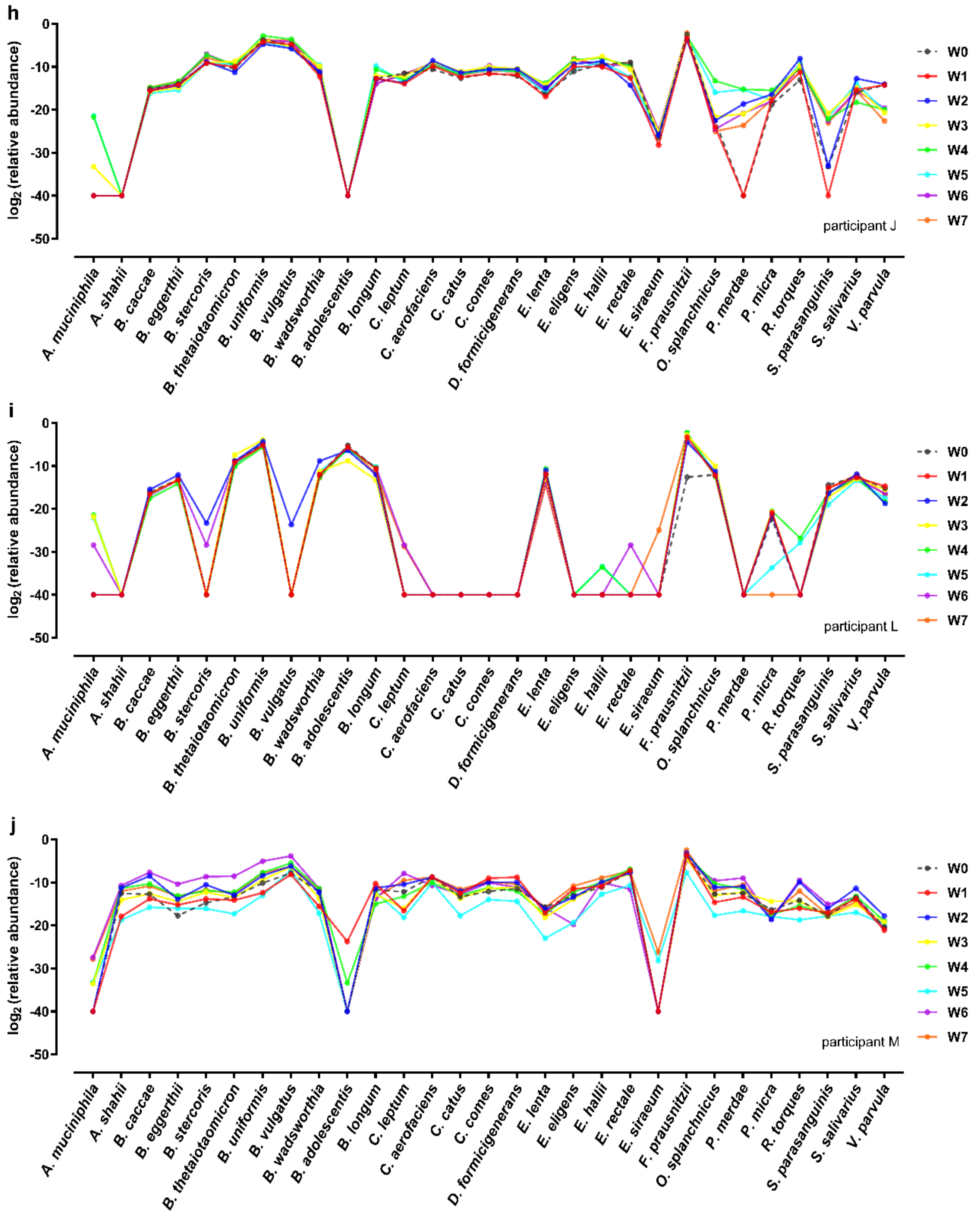

Fig. S2 Dynamic changes in the species abundance of each target bacteria for the 14 participants. The x-axis represents the 29 selected core microbes. The y-axis [$\log_2$ (relative abundance)] represents the relative abundance of these 29 bacteria. The samples were collected from 14 participants once a week for 8 weeks

Table S1 Selection criteria for the 45 gut core microbes

No.	Species	Experiment Strains	Selection Criteria
1	*Akkermansia muciniphila*	[a] ATCC BAA-835	[1] [2] [6] [7]
2	*Alistipes shahii*	[b] BIME 1191250	[1] [3] [5] [6]
3	*Bacteroides caccae*	BIME 1190883	[1] [3] [4] [6] [7]
4	*Bacteroides dorei*	BIME 1100298	[1] [4] [6] [7]
5	*Bacteroides eggerthii*	BIME 1191590	[1] [3] [4] [6] [7]
6	*Bacteroides fragilis*	BIME 1190664	[1] [3] [4] [6] [7]
7	*Bacteroides stercoris*	BIME 1100005	[1] [3] [4] [6] [7]
8	*Bacteroides thetaiotaomicron*	BIME 1100297	[1] [4] [6] [7]
9	*Bacteroides uniformis*	BIME 1100008	[1] [3] [4] [5] [6] [7]
10	*Bacteroides vulgatus*	BIME 1100007	[1] [4] [5] [6] [7]
11	*Bifidobacterium adolescentis*	BIME 1191119	[1] [3] [7]
12	*Bifidobacterium longum*	BIME 1100004	[1] [3] [7]
13	*Bilophila wadsworthia*	BIME 1503827	[1] [2] [6]
14	*Clostridium bolteae*	BIME 1191010	[1] [3] [7]
15	*Clostridium butyricum*	BIME 1191138	[2] [6]
16	*Clostridium leptum*	ATCC 29065	[1] [3] [4] [6] [7]
17	*Clostridium ramosum*	BIME 1192289	[1] [6]
18	*Clostridium saccharolyticum*	ATCC 35040	[3] [7]
19	*Collinsella aerofaciens*	BIME 1191121	[1] [3] [4] [7]
20	*Coprococcus catus*	ATCC 27761	[3] [6]
21	*Coprococcus comes*	ATCC 27758	[1] [3] [4] [6] [7]
22	*Dorea formicigenerans*	ATCC 27755	[1] [3] [4] [7]
23	*Eggerthella lenta*	BIME 1100065	[3] [6] [7]
24	*Enterococcus faecalis*	BIME 1100003	[3] [4]
25	*Eubacterium eligens*	ATCC 27750	[1] [3] [7]
26	*Eubacterium hallii*	ATCC 27751	[3] [4] [6] [7]
27	*Eubacterium limosum*	BIME 1100036	[1] [3]
28	*Eubacterium rectale*	ATCC 33656	[1] [3] [4] [5] [6] [7]
29	*Eubacterium siraeum*	ATCC 29066	[1] [3] [4] [6] [7]
30	*Faecalibacterium prausnitzii*	ATCC 27768	[3] [4] [5] [6] [7]
31	*Gemella morbillorum*	BIME 1502088	[3]
32	*Lactobacillus ruminis*	[c] DSM 20403	[3] [7]

33	*Odoribacter splanchnicus*	BIME 1192728	[1] [3] [6]
34	*Parabacteroides distasonis*	BIME 1100006	[1] [4] [6] [7]
35	*Parabacteroides merdae*	BIME 1100038	[1] [3] [4] [6] [7]
36	*Parvimonas micra*	BIME 1502111	[2]
37	*Peptostreptococcus anaerobius*	ATCC 27337	[3]
38	*Porphyromonas asaccharolytica*	ATCC 25260	[3]
39	*Prevotella melaninogenica*	BIME 1502684	[2] [6]
40	*Ruminococcus bromii*	ATCC 27255	[1] [3] [4] [6]
41	*Ruminococcus gnavus*	ATCC 29149	[1] [3] [4] [6] [7]
42	*Ruminococcus torques*	ATCC 27756	[1] [3] [4] [7]
43	*Streptococcus parasanguinis*	BIME 1502022	[1] [3]
44	*Streptococcus salivarius*	BIME 1503401	[1] [3]
45	*Veillonella parvula*	BIME 1502010	[1]

[a] ATCC (American Type Culture Collection, USA). [b] BIME (Beijing Institute of Microbiology and Epidemiology, China). [c] DSM (Deutsche Sammlung von Mikroorganismen und Zellkulturen, Germany).

[1] Species of relative abundance (in at least one sample) ≥1% and prevalence ≥10% (Tramontano et al. 2018)

[2] One of 316 core species present in at least one sample (4 samples) from 75 individuals (Olsson et al. 2022)

[3] One of 184 core species that were present in all 300 samples from 75 individuals (Olsson et al. 2022)

[4] One of the most common 57 species present in ≥90% of individuals (n=124) with genome coverage >1% (Qin et al. 2010)

[5] Present in more than 95% of 8,208 individuals (Gacesa et al. 2022)

[6] The core species shared by dominant and/or common species (Liu et al. 2021)

[7] The most prevalent organisms from metagenomic sequence data of the NIH Human Microbiome Project (HMP) (Cheng et al. 2022)

Table S2 Bacterial strains used for the specificity test

No.	Species	Experiment Strains
1	*Actinomyces odontolyticus*	BIME 1502011
2	*Bacteroides fragilis enterotoxigenic*	ATCC 43860
3	*Bacteroides ovatus*	BIME 1100009
4	*Blautia hansenii*	ATCC 27752
5	*Clostridium difficile*	ATCC BAA-1382
6	*Escherichia coli*	DSM 682
7	*Fusobacterium nucleatum*	BIME 1502062
8	*Lactabacillus delbrueckii*	ATCC 9649
9	*Lactobacillus plantarum*	BIME 1700312
10	*Lactobacillus acidophilus*	ATCC 4356
11	*Lactobacillus fermentum*	ATCC 14931
12	*Lactobacillus gasseri*	ATCC 33323
13	*Lactobacillus paracasei*	DSM 5622
14	*Lactobacillus sakei*	ATCC 33323
15	*Lactobacillus salivarius*	DSM 20555
16	*Lactobacillus vaginalis*	DSM 5837
17	*Parabacteroides goldsteinii*	BIME 1190197
18	*Solobacterium moorei*	BIME 1507072
19	*Streptococcus gallolyticus*	BIME 1400439

Table S3 LOD, linearity, and primer coverage rate of the 45 qPCR assays

No.	Species	LOD	Linearity Equation	R^2	[a] Coverage
1	*Akkermansia muciniphila*	0.1 pg/μL	y = -4.47x+17.60	0.9898	85.96%
2	*Alistipes shahii*	0.1 pg/μL	y = -3.69x + 16.35	0.9979	91.67%
3	*Bacteroides caccae*	0.1 pg/μL	y = -4.77x + 15.74	0.9929	70.00%
4	*Bacteroides dorei*	0.1 pg/μL	y = -4.61x + 14.42	0.9887	80.00%
5	*Bacteroides eggerthii*	0.1 pg/μL	y = -4.85x + 15.15	0.9955	100.00%
6	*Bacteroides fragilis*	1 pg/μL	y = -5.10x + 19.02	0.9703	89.24%
7	*Bacteroides stercoris*	1 pg/μL	y = -4.60x + 17.95	0.9942	94.03%
8	*Bacteroides thetaiotaomicron*	1 pg/μL	y = -4.55x + 19.56	0.9932	100.00%
9	*Bacteroides uniformis*	1 pg/μL	y = -5.53x + 16.34	0.9885	100.00%
10	*Bacteroides vulgatus*	0.1 pg/μL	y = -4.55x + 15.06	0.9988	73.33%
11	*Bifidobacterium adolescentis*	1 pg/μL	y = -4.75x + 17.51	0.9858	100.00%
12	*Bifidobacterium longum*	0.1 pg/μL	y = -4.32x + 17.52	0.9982	99.54%
13	*Bilophila wadsworthia*	1 pg/μL	y = -5.16x + 18.88	0.9971	[b] -
14	*Clostridium bolteae*	0.1 pg/μL	y = -4.84x + 14.86	0.9962	100.00%
15	*Clostridium butyricum*	0.1 pg/μL	y = -4.66x + 17.37	0.9911	96.43%
16	*Clostridium leptum*	0.1 pg/μL	y = -4.59x + 16.74	0.9993	100.00%
17	*Clostridium ramosum*	1 pg/μL	y = -5.29x + 17.42	0.9932	94.74%
18	*Clostridium saccharolyticum*	1 pg/μL	y = -4.82x + 18.10	0.9952	25.00%
19	*Collinsella aerofaciens*	1 pg/μL	y = -5.16x + 16.89	0.9817	73.64%
20	*Coprococcus catus*	1 pg/μL	y = -5.28x + 16.67	0.9857	100.00%
21	*Coprococcus comes*	1 pg/μL	y = -5.02x + 17.18	0.9927	96.23%
22	*Dorea formicigenerans*	1 pg/μL	y = -6.03x + 16.26	0.981	100.00%
23	*Eggerthella lenta*	1 pg/μL	y = -5.34x + 18.68	0.9968	100.00%
24	*Enterococcus faecalis*	0.1 pg/μL	y = -4.39x + 17.32	0.9994	99.79%
25	*Eubacterium eligens*	1 pg/μL	y = -4.70x + 17.74	0.9975	91.67%
26	*Eubacterium hallii*	1 pg/μL	y = -4.94x + 17.67	0.9836	100.00%
27	*Eubacterium limosum*	1 pg/μL	y = -4.41x + 17.23	0.999	60.00%
28	*Eubacterium rectale*	0.1 pg/μL	y = -3.73x + 20.52	0.9903	95.00%
29	*Eubacterium siraeum*	1 pg/μL	y = -5.57x + 16.00	0.9966	100.00%
30	*Faecalibacterium prausnitzii*	0.1 pg/μL	y = -4.78x + 14.34	0.9974	81.25%

31	*Gemella morbillorum*	1 pg/µL	y = -6.09x + 15.61	0.9669	100.00%
32	*Lactobacillus ruminis*	1 pg/µL	y = -5.11x + 16.59	0.997	89.29%
33	*Odoribacter splanchnicus*	0.1 pg/µL	y = -3.68x + 17.17	0.999	97.50%
34	*Parabacteroides distasonis*	0.1 pg/µL	y = -4.09x + 18.01	0.9965	99.27%
35	*Parabacteroides merdae*	0.1 pg/µL	y = -3.93x + 16.96	0.9937	100.00%
36	*Parvimonas micra*	0.1 pg/µL	y = -4.75x + 16.94	0.992	100.00%
37	*Peptostreptococcus anaerobius*	1 pg/µL	y = -4.86x + 16.25	0.9943	88.89%
38	*Porphyromonas asaccharolytica*	1 pg/µL	y = -5.36x + 16.13	0.9959	100.00%
39	*Prevotella melaninogenica*	1 pg/µL	y = -5.64x + 17.14	0.992	85.71%
40	*Ruminococcus bromii*	0.1 pg/µL	y = -4.91x + 14.94	0.9948	96.23%
41	*Ruminococcus gnavus*	1 pg/µL	y = -4.38x + 16.74	0.9958	100.00%
42	*Ruminococcus torques*	0.1 pg/µL	y = -4.68x + 15.97	0.9971	75.00%
43	*Streptococcus parasanguinis*	1 pg/µL	y = -4.80x + 17.85	0.9985	100.00%
44	*Streptococcus salivarius*	1 pg/µL	y = -5.73x + 17.49	0.9947	100.00%
45	*Veillonella parvula*	0.1 pg/µL	y = -4.08x + 16.01	0.9976	90.63%

[a] For primers designed in this study, the coverage rate was calculated based on the number of NCBI genomic sequences of corresponding target bacteria, including genomes at various assembly levels (the data was acquired in July 2021); for primers obtained from literature, the coverage rate was calculated based on the number of complete genomes.

[b] The coverage rate was not calculated due to the unavailability of complete genomes of the target bacterium in the NCBI database.

References

Cheng AG, Ho PY, Aranda-Diaz A, Jain S, Yu FB, Meng X, Wang M, Iakiviak M, Nagashima K, Zhao A, Murugkar P, Patil A, Atabakhsh K, Weakley A, Yan J, Brumbaugh AR, Higginbottom S, Dimas A, Shiver AL, Deutschbauer A, Neff N, Sonnenburg JL, Huang KC, Fischbach MA (2022) Design, construction, and in vivo augmentation of a complex gut microbiome. Cell 185(19):3617-3636 e19. http://doi.org/10.1016/j.cell.2022.08.003

Gacesa R, Kurilshikov A, Vich Vila A, Sinha T, Klaassen MAY, Bolte LA, Andreu-Sánchez S, Chen L, Collij V, Hu S, Dekens JAM, Lenters VC, Björk JR, Swarte JC, Swertz MA, Jansen BH, Gelderloos-Arends J, Jankipersadsing S, Hofker M, Vermeulen RCH, Sanna S, Harmsen HJM, Wijmenga C, Fu J, Zhernakova A, Weersma RK (2022) Environmental factors shaping the gut microbiome in a Dutch population. Nature 604(7907):732-739. http://doi.org/10.1038/s41586-022-04567-7

Liu C, Du M-X, Abuduaini R, Yu H-Y, Li D-H, Wang Y-J, Zhou N, Jiang M-Z, Niu P-X, Han S-S, Chen H-H, Shi W-Y, Wu L, Xin Y-H, Ma J, Zhou Y, Jiang C-Y, Liu H-W, Liu S-J (2021) Enlightening the taxonomy darkness of human gut microbiomes with a cultured biobank. Microbiome 9(1):119. http://doi.org/10.1186/s40168-021-01064-3

Olsson LM, Boulund F, Nilsson S, Khan MT, Gummesson A, Fagerberg L, Engstrand L, Perkins R, Uhlén M, Bergström G, Tremaroli V, Bäckhed F (2022) Dynamics of the normal gut microbiota: A longitudinal one-year population study in Sweden. Cell Host Microbe 30(5):726-739.e3. http://doi.org/10.1016/j.chom.2022.03.002

Qin J, Li R, Raes J, Arumugam M, Burgdorf KS, Manichanh C, Nielsen T, Pons N, Levenez F, Yamada T, Mende DR, Li J, Xu J, Li S, Li D, Cao J, Wang B, Liang H, Zheng H, Xie Y, Tap J, Lepage P, Bertalan M, Batto J-M, Hansen T, Le Paslier D, Linneberg A, Nielsen HB, Pelletier E, Renault P, Sicheritz-Ponten T, Turner K, Zhu H, Yu C, Li S, Jian M, Zhou Y, Li Y, Zhang X, Li S, Qin N, Yang H, Wang J, Brunak S, Doré J, Guarner F, Kristiansen K, Pedersen O, Parkhill J, Weissenbach J, Bork P, Ehrlich SD, Wang J (2010) A human gut microbial gene catalogue established by metagenomic sequencing. Nature 464(7285):59-65. http://doi.org/10.1038/nature08821

Tramontano M, Andrejev S, Pruteanu M, Klünemann M, Kuhn M, Galardini M, Jouhten P, Zelezniak A, Zeller G, Bork P, Typas A, Patil KR (2018) Nutritional preferences of human gut bacteria reveal their metabolic idiosyncrasies. Nat Microbiol 3(4):514-522. http://doi.org/10.1038/s41564-018-0123-9
